# 半乳凝素-3在非小细胞肺癌中的表达及相关性研究

**DOI:** 10.3779/j.issn.1009-3419.2013.08.05

**Published:** 2013-08-20

**Authors:** 美燕 刘, 冰 杜, 春红 李, 艳滨 赵, 庆威 孟, 莉 蔡

**Affiliations:** 1 150081 哈尔滨，哈尔滨医科大学附属肿瘤医院内四科 Department of Medicine, Afliated Tumor Hospital of Harbin Medical University, 150081 Harbin, China; 2 150086 哈尔滨，哈尔滨医科大学附属第二医院内科 Department of Medicine, The Second Afliated Hospital of Harbin Medical University, 150086 Harbin, China

**Keywords:** 肺肿瘤, 半乳凝素-3, 凋亡, Lung neoplasms, Galectin-3, Apoptosis

## Abstract

**背景与目的:**

半乳凝素-3（Galectin-3）参与许多生理和病理过程，与肿瘤的发生、发展、转移关系密切。本研究旨在探讨Galectin-3在非小细胞肺癌（non-small cell lung cancer, NSCLC）中的表达情况及其与临床病理特征间的关系，探索其在NSCLC发生、发展中的作用。

**方法:**

采用S-P免疫组化方法检测62例NSCLC组织和17例正常肺组织中Galectin-3的表达情况，并用RT-PCR方法在转录水平进行验证，并结合相关的临床病理资料进行分析。

**结果:**

免疫组化结果显示Galectin-3在83.8%（52/62）的NSCLC组织中阳性表达。NSCLC组织中Galectin-3表达阳性率明显高于正常肺组织（*χ*^2^=7.936, *P* < 0.01），其在癌组织中的表达与患者年龄、性别和病理分型无关。Galectin-3与肿瘤的分化程度（*χ*^2^=8.577, *P* < 0.05）和临床分期（*χ*^2^=5.287, *P* < 0.05）相关，其中与分化程度（*r*=-0.292, *P* < 0.05）的表达呈负相关，与临床分期（*r*=0.336, *P* < 0.05）的表达呈正相关。RT-PCR结果显示，Galectin-3在NSCLC组织中的表达明显高于正常肺组织，随访结果表明Galectin-3高表达者总生存小于低表达者（*P*=0.045)。

**结论:**

Galectin-3可能是肺癌诊断的重要辅助基因，Galectin-3有望与肺癌其它标志物联合检查以提高诊断率，提示患者预后。

## Abstract (en)

**Background and objective:**

Galectin-3 is involved in many physical and pathologic process, and is closely correlated with tumorigenesis, development and metastasis. The aim of this study is to investigate the gene expression and signifcance of Galectin-3 in normal lung tissue and non-small cell lung cancer (NSCLC) tissue.

**Methods:**

Gene expression was examined using the immunohistochemical S-P method in 62 NSCLC cases and in 17 cases with normal lung tissue. RT-PCR verifed the expression of Galectin-3 at the transcriptional level.

**Results:**

The expression rate of Galectin-3 in NSCLC tissue was 83.8% (52/62), which was signifcantly higher than that in normal lung tissue (*P* < 0.01). Galectin-3 expression was not correlated with sex (*χ*^2^=0.113, *P* > 0.05), age (*χ*^2^=0.220, *P* > 0.05), and histological type (*χ*^2^=0.012, *P* > 0.05), but was negatively correlated with pathological differentiation (*r*=-0.292, *P* < 0.05) and positively correlated with the clinical stage (*r*=0.336, *P* < 0.05) in NSCLC tissue. RT-PCR results showed that the expression level of Galectin-3 in NSCLC tissue was signifcantly higher than in normal lung tissue.

**Conclusion:**

Galectin-3 may be useful in lung cancer diagnosis and may increase diagnosis rate when combined with other lung cancer markers.

半乳凝素-3(Galectin-3)能与β-半乳糖苷特异性结合，参与许多生理和病理过程，在调解机体免疫、细胞粘附、炎症反应、细胞凋亡、血管侵袭等诸多方面起着重要的作用^[1]^，且与肿瘤的转移及预后有关。目前，在许多恶性肿瘤中都发现了Galectin-3的表达，但在不同的肿瘤中，Galectin-3的表达水平不同。在肺癌中的研究比较少。

本实验运用免疫组化法和RT-PCR方法，结合完整的临床资料检测Galectin-3在非小细胞肺癌(non-small cell lung cancer, NSCLC)中的表达，分析Galectin-3的表达与NSCLC发生发展的关系，并探讨其表达与临床病理特征之间的关系。

## 1 材料与方法

### 1.1 材料

17例正常肺组织取于哈尔滨医科大学附属肿瘤医院2006年10月-2007年2月液氮冻存组织，52例NSCLC组织标本取自哈尔滨医科大学附属肿瘤医院2006年1月-2007年5月手术切除病理存档蜡块，52例组织均有良好随访记录(> 50个月)，所有手术患者术前均未接受过放疗、化疗，诊断均经常规病理切片证实。组织均经甲醛固定，石蜡包埋，厚4 μm连续切片，分别作HE和免疫组化染色。10例NSCLC组织为哈尔滨医科大学附属肿瘤医院2006年11月-2007年2月手术切除的新鲜肺癌组织，所有原发肿瘤标本在切除后被立即分成两部分：一部分经甲醛固定、石蜡包埋用于免疫组化检测，另一部分储存于液氮中用于RT-PCR检测。

### 1.2 临床及病理资料

62例NSCLC按国际抗癌联盟2009年修订的TNM分期标准分为：Ⅰa期-Ⅱb期29例，Ⅲa期-Ⅲb期33例；根据2011年WHO公布的肺癌组织分类标准判定病理类型和分化程度。肺腺癌32例，肺鳞癌30例；高分化24例，中分化17例，低分化21例；男性34例，女性28例；年龄从35岁-79岁，中位年龄为57.1岁。10例新鲜非小细胞肺癌组织中鳞癌6例，腺癌4例。

### 1.3 实验试剂与方法

#### 1.3.1 S-P法

一抗Galectin-3(NCL-GAL3)鼠单抗为Santa Cruz公司产品，Ultrasensitive^TM^ S-P免疫组织化学染色试剂盒购自福州迈新生物技术开发公司。DAB显色剂购自福州迈新生物技术开发公司。免疫组化染色程序按S-P试剂盒说明书进行，一抗Galectin-3羊多抗(1:50)，每批染色均设立阴性空白对照(以PBS液代替一抗)。

#### 1.3.2 RT-PCR法

TaKaRa RNA PCR试剂盒购自大连宝生物公司；RNA提取试剂Trizol购自Inv itrogen公司。DL2000 DNA Marker购自大连宝生物公司。RNA一步法提取总RNA，紫外分光光度计测量样品浓度和纯度，所得RNA_260/280_值在1.8-2.0，电泳18S和28S RNA条带清晰。按Oligotex mRNA Midi Kit(MagExtractor公司)说明分离纯化为mRNA。各引物均由大连宝生物合成，扩增条件：94 ℃、2 min，94 ℃、30 s，56 ℃、30 s，72 ℃、30 s。30个循环。RT-PCR所用的引物序列如下：Galectin-3上游：5'-ATGGCAGACAATTTTTCGCTCC-3'；下游：5'-ATGTCACCAGAAATTCCCAGTT-3'；β-actin上游：5'-TGACGGGGTCACCCACACTGTGCCCATCTA-3'；下游：5'-CTAGAAGCATTGCGGTGGACGATGGAGGG-3'。

### 1.4 免疫组化结果

分别由两名经验丰富的病理医师在不知临床和病理资料的情况下独立观察切片，对免疫组化结果进行评估。Galectin-3主要表达于胞质和胞核中，阳性表达为黄色或黄棕色颗粒。按染色强度及染色细胞数量划分等级，阴性(-)：显色强度与背景无明显差别；弱阳性(+)：显色强度为淡黄色或仅个别细胞呈黄至棕黄色染色，阳性细胞数 < 10%；中度阳性(++)：显色强度呈黄至棕黄色染色，阳性细胞10%-60%；强阳性(+++)：显色强度呈棕褐色，阳性细胞数 > 60%。

### 1.5 随访

采用电话回访和病案查询的方式进行随访，记录总生存期(overall survival, OS)。OS定义为从术后第一天至死亡或随访截止时间。随访截止于2012年12月31日。

### 1.6 统计学方法

采用SPSS 19.0软件，对数据进行*χ*^2^检验，非参数秩和检验及*Spearman*等级相关检验，*P* < 0.05有统计学差异。

## 2 结果

### 2.1 Galectin-3在NSCLC组织中的表达

Galectin-3在正常肺组织阳性表达率为11.76%(2/17)，在NSCLC组织中阳性表达率为83.8%(52/62)，二者差异有统计学意义(*χ*^2^=30.069, *P* < 0.001)，即NSCLC组织中Galectin-3的阳性表达率明显高于正常肺组织(图 1)。

**1:**
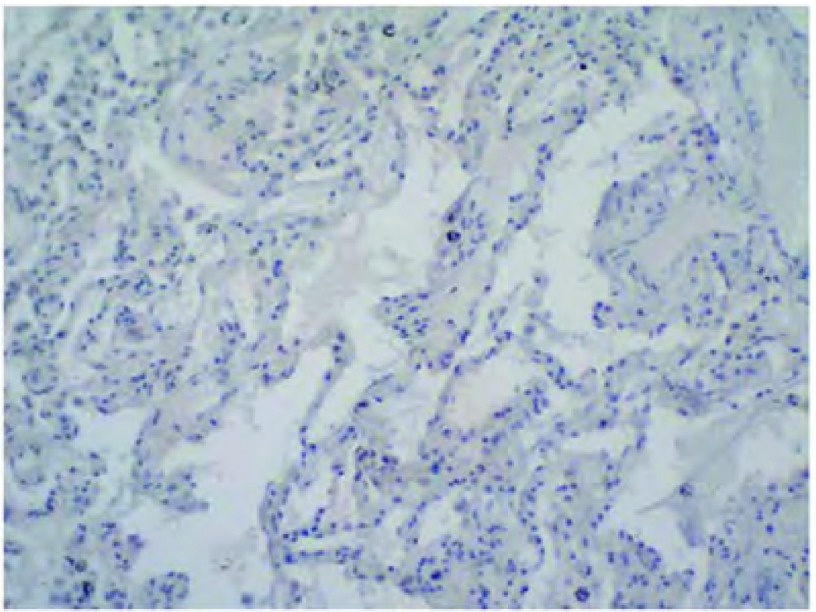
Galectin-3在正常肺组织的阴性表达（S-P法，×200） The negative expression of Galectin-3 in lung tissue(S-P method, ×200)

### 2.2 Galectin-3的表达与临床病理特征的关系

Galectin-3在NSCLC组织中的表达与患者的性别(*χ*^2^=0.113, *P* > 0.05)和年龄(*χ*^2^=0.220, *P* > 0.05)差异无统计学意义(表 1)。Galectin-3在肺鳞癌中阳性率为86.2%(27/32)，肺腺癌中阳性率为80.8%(25/30)，二者之间无统计学差异(*χ*^2^=0.012, *P* > 0.05)(图 2)；Galectin-3与肿瘤的分化程度(*χ*^2^=8.577, *P* < 0.05)和临床分期(*χ*^2^=5.287, *P* < 0.05)相关，其中与分化程度(*r*=-0.292, *P* < 0.05)的表达呈负相关，与临床分期(*r*=0.336, *P* < 0.05)的表达呈正相关。

**1:** Galectin-3在非小细胞肺癌组织中的表达及与临床病理特征的关系 Relationship between the expression of Galectin-3 in non-small cell lung cancer tissue and clinicopathological features

Characteristic	*n*	Galectin-3				
		-	+	Positive (%)	*χ*^2^	*P*
Sex					0.113	0.737
Male	34	5	29	85.3		
Female	28	5	23	82.1		
Age (yr)					0.22	0.639
≤50	33	6	27	81.8		
> 50	29	4	25	86.2		
Histological					0.012	0.911
SCC	32	5	27	86.2		
AC	30	5	25	80.8		
Degree of differentiation					8.577	0.014
Well differentiation	24	8	16	66.7		
Moderate differentiation	17	1	16	94.1		
Poor differentiation	21	1	20	95.2		
p-TNM status					5.287	0.021
Ⅰa-Ⅱb	29	8	21	72.4		
Ⅲa-Ⅳb	33	2	31	93.9		
SCC: squamous cell carcinoma; AC: adenocarcinoma.						

**2:**
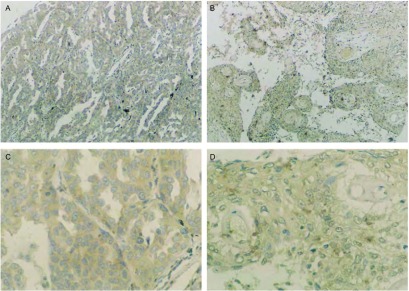
Galectin-3在肺腺癌和肺鳞癌中呈阳性表达（S-P法，A，B：×100；C，D：×400） The expression of Galectin-3 in lung adenocarcinoma and squamous cell carcinoma (S-P method, A, B: ×100; C, D: ×400)

### 2.3 RT-PCR中Galectin-3的表达水平

RT-PCR结果表明在10例新鲜NSCLC组织中无论是鳞癌组织还是腺癌组织Galectin-3均为高表达，其阳性表达率明显高于正常肺组织(图 3)。这与免疫组化的结果是一致的，进一步证实了免疫组化的结果。

**3:**
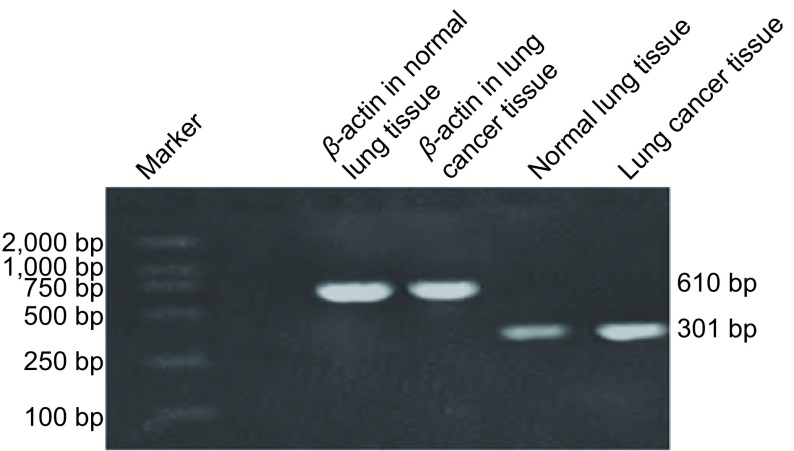
RT-PCR结果：Galectin-3在非小细胞肺癌组织中表达明显高于正常肺组织 Results of RT-PCR: Expression of Clusterin in lung cancer tissues was significantly higher than in normal lung tissues

### 2.4 Galectin-3的表达与患者生存率的关系

Galectin-3阳性表达的肺癌患者中位生存期为34.56个月，Galectin-3阴性表达的肺癌患者中位生存期 > 80个月，存在统计学差异(*P*=0.045)(图 4)。

**4:**
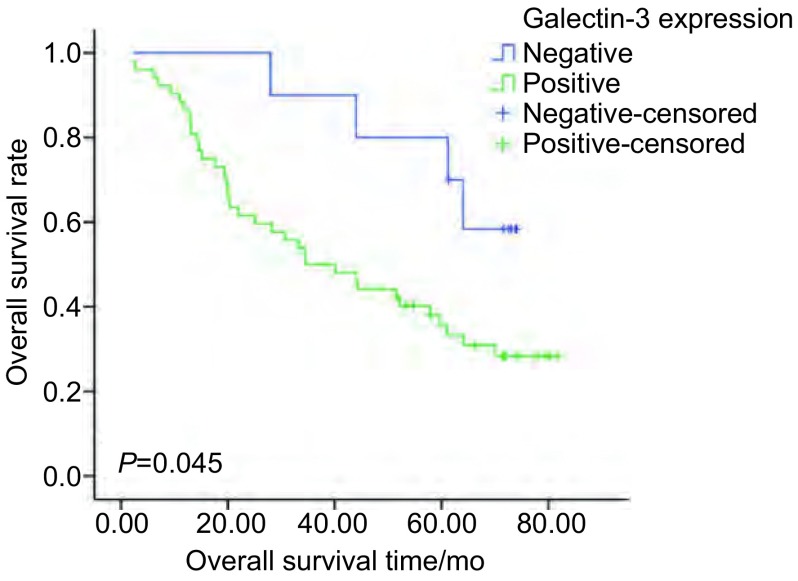
Galectin-3与总生存期的关系 The realationship between Galectin-3 and overall survival

## 3 讨论

Galectins是凝集素家族中的一员，能与β-半乳糖苷特异性结合。自1975年报道第一个哺乳动物Galectin以来，现已发现15个成员^[2]^。其中对Galectin-3的研究最为广泛，它首先发现于巨噬细胞中，现在发现一些其它组织中也存在，如：肠、脑、肾、骨、肺、前列腺^[3-5]^及许多癌组织中。

Galectin-3是半乳糖凝集素家族的重要成员之一，与细胞内的糖蛋白、细胞表面分子和细胞外基质作用，参与细胞粘附、增殖、凋亡、血管形成及mRNA剪切^[1, 6-7]^等，与许多肿瘤的细胞增殖及侵袭、转移等恶性生物学行为密切相关。目前，在许多恶性肿瘤中都发现了Galectin-3的表达，如乳腺癌、前列腺癌、甲状腺癌、肝癌、星形胶质细胞瘤等^[8-11]^。Galectin-3不仅与肿瘤的发生有关，与肿瘤的转移也有关联^[11]^。已报道Galectin-3在多种肿瘤中过表达并参与肿瘤转移的调控，包括胃癌、脑肿瘤、结肠癌和甲状腺癌^[12, 13]^。

肺癌是当今世界上严重威胁人类生命健康的恶性肿瘤之一，发病率在许多国家都有增高趋势，根据WHO的数据，肺癌目前是全世界癌症死因的第一名，约占全部恶性肿瘤的19%。全世界每年的新增病例超过120万。在男性肿瘤死因中已居首位，在女性中仅次于乳腺癌居第二位。因此，早期诊断和治疗极为重要。Mathieu等于2003年首次报道了*Galectin*-3基因在肺癌中的表达情况，其中NSCLC组中*Galectin*-3基因过表达，表达量是正常上皮细胞的3倍；相反，小细胞肺癌(small cell lung cancer, SCLC)者均不表达或低表达。此研究^[14]^还证实过表达的Galectin-3表达水平上调可能促进了NSCLC的转移，但与SCLC转移无关。Buttery等用免疫组化法对不同类型肺癌组织中Galectin-3的表达进行评估。证实在NSCLC中，Galectin-3呈高表达；而在SCLC中，Galectin-3表达水平及低。在NSCLC中，鳞癌与腺癌中Galectin-3的表达没有明显区别，且均表达在胞浆和胞核中^[15]^。在鳞癌细胞中，Galectin-3在不同分化程度的细胞中表达水平不同，肿瘤分化程度越低其表达水平越高，但差别没有统计学意义。在腺癌细胞中，Galectin-3在不同分化程度的细胞中表达水平未见明显不同^[15]^。

为了进一步观察Galectin-3在NSCLC中的表达与临床病理特征间的关系，为临床诊断和预后寻找新的靶点，我们选取了62例NSCLC组织和17例正常肺组织进行了免疫组化方法检测。结果显示：NSCLC组织中Galectin-3的表达明显高于正常肺组织，提示Galectin-3在NSCLC的发生发展中可能起一定的意义，与Buttery等的研究结果一致。另外我们发现NSCLC中不同的TNM分期、不同的病理分级与Galectin-3的表达具有相关性，癌组织的分化越差、分期越晚Galectin-3表达越高(*P* < 0.05)，RT-PCR的结果进一步证实Galectin-3在肺癌组织中的表达明显高于正常肺组织，说明其表达高低与肿瘤的恶性生物学行为有关。随访结果亦显示Galectin-3与患者的总生存率相关，Galectin-3阳性表达的患者预后较差(*P*=0.045)。由此可见，Galectin-3可能是评价NSCLC预后的又一个重要基因，可能作为判断NSCLC恶性程度及不良预后的生物学指标之一。

生物学标志物的不断发现，为NSCLC的诊断和治疗提供了重要线索。Galectin-3有望成为今后治疗NSCLC的一个新靶点，与肺癌其它标志物联合检查以提高诊断率。总之，我们通过免疫组化方法检测NSCLC组织及正常肺组织中Galectin-3蛋白的表达情况，分析发现Galectin-3蛋白的表达与患者的临床特征及预后密切相关，对判断NSCLC患者的预后具有重要意义。
